# Potential Regulation of miRNA-29 and miRNA-9 by Estrogens in Neurodegenerative Disorders: An Insightful Perspective

**DOI:** 10.3390/brainsci13020243

**Published:** 2023-01-31

**Authors:** Mahmoud A. Ebada, Abdalrhman Mostafa, Al-Hussein A. Gadallah, Souad Alkanj, Badrah S. Alghamdi, Ghulam Md Ashraf, Adel M. Abuzenadah, Raed F. Alserihi, Ahmed Wadaa-Allah, Mohamed Salama

**Affiliations:** 1 Neurologist, Nasr City Hospital for Health Insurance, Cairo 11765, Egypt; 2 Resident, Egyptian Fellowship of Neurology, Ministry of Health and Population of Egypt, Cairo 4262124, Egypt; 3 Faculty of Medicine, Zagazig University, Zagazig 44519, Egypt; 4 Intern Physician, Mansoura University Hospitals, Dakahlia 7650030, Egypt; 5 Resident at the Egyptian Fellowship Program of Clinical Pathology, Assuit 71515, Egypt; 6 Neuroscience Unit, Department of Physiology, Faculty of Medicine, King Abdulaziz University, Jeddah 21589, Saudi Arabia; 7 Pre-Clinical Research Unit, King Fahd Medical Research Center, King Abdulaziz University, Jeddah 21589, Saudi Arabia; 8 Department of Medical Laboratory Sciences, College of Health Sciences, Sharjah Institute for Medical Research, University of Sharjah, Sharjah P.O. Box 27272, United Arab Emirates; 9 Department of Medical Laboratory Sciences, Faculty of Applied Medical Sciences, King Abdulaziz University, Jeddah 21589, Saudi Arabia; 10 King Fahd Medical Research Center, King Abdulaziz University, Jeddah 21859, Saudi Arabia; 11 Hematology Research Unit, King Fahd Medical Research Center, King Abdulaziz University, Jeddah 21589, Saudi Arabia; 12 Nanomedicine Unit, Center of Innovation in Personalized Medicine, King Abdulaziz University, Jeddah 21589, Saudi Arabia; 13 Biochemistry Department, Faculty of Science, Ain Shams University, Cairo 4392001, Egypt; 14 Institute of Global Health and Human Ecology, the American University in Cairo, Cairo 4728120, Egypt

**Keywords:** miRNA-9, miRNA-29, regulation, compounds, estrogenic

## Abstract

Finding a link between a hormone and microRNAs (miRNAs) is of great importance since it enables the adjustment of genetic composition or cellular functions without needing gene-level interventions. The dicer-mediated cleavage of precursor miRNAs is an interface link between miRNA and its regulators; any disruption in this process can affect neurogenesis. Besides, the hormonal regulation of miRNAs can occur at the molecular and cellular levels, both directly, through binding to the promoter elements of miRNAs, and indirectly, via regulation of the signaling effects of the post-transcriptional processing proteins. Estrogenic hormones have many roles in regulating miRNAs in the brain. This review discusses miRNAs, their detailed biogenesis, activities, and both the general and estrogen-dependent regulations. Additionally, we highlight the relationship between miR-29, miR-9, and estrogens in the nervous system. Such a relationship could be a possible etiological route for developing various neurodegenerative disorders.

## 1. Introduction

Non-coding RNAs (ncRNAs) include miRNAs, short-interfering RNAs (siRNAs), and PIWI-interacting RNA (piRNAs) [1]. MiRNAs are small single-stranded, non-coding RNAs that are 22–23 nucleotides in length. More than 2000 miRNA genes were identified [2]. The primary function of miRNAs is to regulate gene expression, each by binding to their specific messenger RNA (mRNA) through its 3’-untranslated region complementary sequence, which leads to degradation of the mRNA or blockage of translation [3].

miRNAs were first discovered in 1993 by Ambros and Ruvkun, who found the Lin-4 miRNA in Caenorhabditis elegans and revealed that it is the first gene in the pathways directing cell division and regulating the process of differentiation of hypodermal stem cells into skin cells [4]. Since then, thousands of miRNAs have been discovered in most living organisms, even in plants and viruses, and their roles in numerous functional processes such as apoptosis, immunity, and metabolism are now under further research [5].

The association between the different miRNAs’ expression and androgens has been confirmed in prostate and breast cancers [6,7,8]. Similarly, sexual miRNA expression panels in the brain have been identified in both diseased and normal vertebrates and non-vertebrates [9,10]. Studies have shown a potential effect of sex chromosomes on miRNA expression [9,11,12,13,14,15].

Sex steroids are mainly synthesized in the gonads. They are also formed in the central nervous system, hence the name “neurosteroids” [16]. Three primary estrogenic steroidal hormones have been identified: estradiol (E2), estrone (E1), and estriol (E3). These have pivotal roles in the processes of fertility, development, and homeostasis in most tissues, including the brain, breast, cardiovascular system, skin, lung, and reproductive tract, both in women and men [17,18,19,20]. 

Steroidal hormones regulate the expression of miRNA through nuclear receptor-mediated pathways [21,22]. E2, the primary circulating type of estrogen in premenopausal women, is formed from cholesterol in the granulosa cells in the ovary due to the stimulating effect of luteinizing hormone (LH) [23], while E1 is the primary estrogen in women after menopause. E1 is synthesized primarily in adipose tissue from the adrenal androgens in a reaction catalyzed by aromatase enzymes [24]. These enzymes are found in neurons and astrocytes [25]. The relationship between miRNAs and estrogens in the context of neurodegenerative disease requires further understanding. Here, we comprehensively review miRNAs with a focus on miR-29 and miR-9′s functions and highlight their interactions with estrogens and various neurodegenerative disorders.

## 2. Estrogens in Brain Development and Function

Sex hormones like estradiol, testosterone, and progesterone regulate miRNA expression by binding to nuclear receptors to alter gene expression directly or indirectly [26]. The brain is both a producer and a target of steroids. Astrocytes form E2 through an aromatase-instigated reaction in many human brain regions, such as the hippocampus, thalamus, and hypothalamus. In stressful conditions (e.g., epilepsy), the estrogen-synthesizing enzyme expression was found to be distorted [27]. Besides, there are widespread estrogen receptors in brain areas, such as the hippocampus, and a high level of responsiveness in these regions to sex hormones, including E2 [28]. Accordingly, E2 is expected to be significantly involved in many physiological processes in the brain, including cognition and neural development [29]. 

The previous literature documented that E2 synthesized in the brain has a core function in synaptogenesis, adjusts the spine’s density, and endorses long-term potentiation [30]. Such observations might have paved the way for some therapeutic applications of E2. For instance, a study demonstrated that E2 injections after a stroke or a brain injury serve neuroprotection, stimulate neurogenesis to generate replacement neurons, and decrease cell death by enhancing neurotrophin support and suppressing pro-inflammatory pathways [31]. Moreover, ovariectomy of middle-aged female rats altered the transcriptome of the hippocampus, which led to a reduction in neurogenesis [32]. 

A previous study has reported that E2 and progesterone treatment of female rats that had undergone ovariectomy improved spatial reference memory and exerted an antidepressant effect through a rise in the turnover rate of monoamines like serotonin. With age, neurogenesis rates decline in rodents and humans due to a decline in the dividing cell number and an associated decline in gonadal hormone levels [33,34]. 

One of the most relevant functions of E2 in the brain is neuroprotection. Many studies have suggested the possible role of E2 in protecting the neural tissue against degenerative events observed in pathologies like Parkinson’s and Alzheimer’s [35]. This can be seen in the late onset of symptoms of Parkinson’s in women. In another context, more severe symptoms of Alzheimer’s were found in postmenopausal women compared to men of the same age group [36,37,38]. However, some contradictory results suggest that the cumulative estrogenic effect during a woman’s fertile life could significantly reduce the severity of the neurodegeneration-related symptoms, thus preserving the observed sex-dependent differences in disease prevalence and life expectancy, which persist even after the age of menopause [39]. 

## 3. miRNA Processing

**Figure 1:**
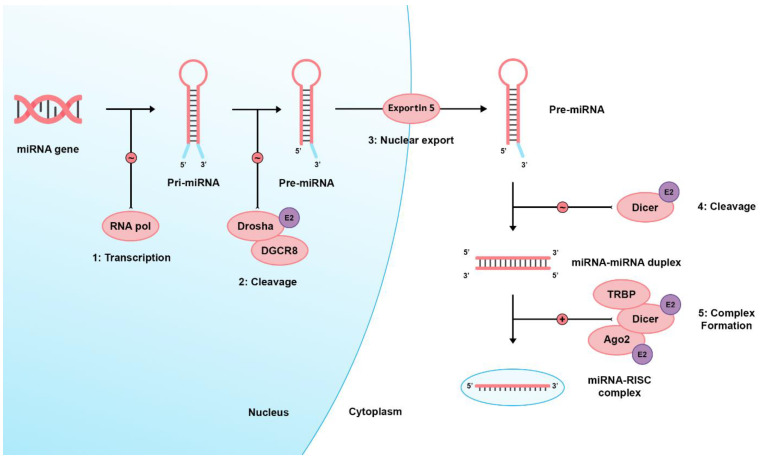
Shows the miRNA biogenesis. Firstly, a large primary (pri-) miRNA is transcribed by RNA polymerase II then pri-miRNA is cleaved by the RNase III enzyme Drosha and coupled with the microprocessor complex subunit DGCR8 to produce pre-miRNA. Pre-miRNAs range from 70 to 90 nucleotides in length and contain a stem-loop structure for their transport to the cell cytoplasm by Exportin-5. In the cytoplasm, this hairpin structure is cleaved by the RNase III enzyme (Dicer), producing the double-stranded miRNA: miRNA duplex. Eventually, a mature miRNA strand is incorporated into a miRNA-associated RNA-induced silencing complex (miRISC), which targets complementary mRNA sequences, producing its cellular effects via transcriptional cleavage or repression. RNA pol: RNA polymerase II, DGCR8: DiGeorge Syndrome Critical Region 8, TRBP: transactivating response RNA-binding protein, Ago2: Argonaute 2, E2: Estrogen 2 signaling, ∼ icon: catalytic process, + icon: complex formation.

There are two pathways regarding miRNA biogenesis: the canonical and noncanonical pathways [40]. In the dominant canonical pathway, miRNA processing starts with RNA polymerase II, transcribing miRNA from the genome into long precursor molecules called primary miRNAs (pri-miRNA). The pri-miRNAs are cleaved by the ribonuclease (RNAase) III family enzyme, Drosha, into precursor miRNAs (pre-miRNAs). Afterward, they form a microprocessor complex by binding with the DiGeorge Syndrome Critical Region 8 (DGCR8), an RNA-binding protein. Then, it is transported to the cytoplasm through active transport via the Ran-GTP-dependent dsRNA-binding protein, Exportin-5, to be processed by the Dicer enzyme (an RNAase III endonuclease), the transactivating response RNA-binding protein (TRBP), and the Kinase R-activating protein. Mature miRNAs are then formed and loaded on an RNA-induced silencing complex (RISC) consisting of Dicer, TRBP, the Kinase R-activating protein, and Argonaute 2 (Ago2). Eventually, they bind to the 3′UTR of target genes, inducing mRNA degradation or translational inhibition [1,41,42,43,44,45]. E2 signaling can alter Ago2, Drosha, and Dicer’s expression [46]. Figure 1 shows a brief and simplified illustration of miRNA biogenesis.

## 4. miRNA Action

miRISC and target mRNA have been detected in several organelles, including lysosomes, rough endoplasmic reticulum, stress granules, processing (P)-bodies, early/late endosomes, trans-Golgi network multivesicular bodies, mitochondria, and the nucleus. Their gene regulatory activity keeps gene expression in a steady state. The availability of miRNAs and their target mRNAs contributes to electing specific genes to be regulated [47]. About 60% of all protein-coding genes in mammals are under miRNA regulation [48]. The dysregulation of miRNAs leads to the disarray of their target genes. Such disruption is clinically correlated to pathological conditions, including neurological disorders, cardiovascular diseases, and tumors [49]. Cytoplasmic miRISC can diffuse through the cytosol or undergo shuttling via microtubules. After combining with polysomes in the cytosol, miRISC can cause mRNA decline, inhibit translation initiation, or improve translational activation. The rough endoplasmic reticulum is usually where translation inhibition occurs through the interaction with target mRNA [50]. Similar processes take place in other organelles [e.g., P-bodies, mitochondria] with the aid of organelle-specific proteins (TNRC6A in the case of P-bodies) [51,52]. The next steps involve the decoupling with Ago2, and the deadenylation and decapping of target mRNA, which have been posited to take place in multivesicular bodies and endosomes [53]. miRISC then has two options: go back to the cytosol or degrade after being shuttled to lysosomes.

miRNA’s actions are not exclusively autocrine; they can have paracrine and endocrine effects with the help of exosomes and other extracellular vesicles [54]. Exosomes are 40 to 100-nanometer membrane-bound vesicles containing different growth factors, cytoplasmic proteins, cytokines, lipids, and nucleic acids (including miRNAs). They circulate in the lymph and blood to dispense those molecules to tissues, thereby mediating the paracrine and endocrine effects. Exosomes can affect tumor progression via factors that promote proliferation, invasion, drug resistance, and metastasis. In this context, circulating miRNAs in exosomes control cellular mechanisms in recipient cells [55]. Extracellular vesicles were generally shown to facilitate miRNA’s endocrine effects related to immunity [56,57,58], endothelial physiology [59], cell differentiation [60,61], synaptic plasticity [62], neural trauma response [63], mitophagy [64], and follicular maturation [65]. 

## 5. miRNA General Regulation

Hormonal regulation of miRNAs can occur at the molecular and cellular levels, both directly and indirectly. It occurs directly when hormones bind to the promoter elements of miRNAs and indirectly when they regulate post-transcriptional processing proteins’ signaling [66]. A tightly balanced and regulated ratio between Drosha and DGCR8 is always achieved in the microprocessor complex: DGCR8 stabilizes Drosha cleaves and inactivates it, generating a stringent feedback loop [67].

Estrogen receptor α (ERα) interacts with p68 to inhibit Drosha complex formation, leading to suppressing pri-miRNA processing [68]. Dicer processes pre-miRNA to mature miRNA. Dicer activity is enhanced by MAPK-phosphorylation (mitogen-activated protein kinase) of TRBP (TAR RNA-binding protein), which promotes miRNA processing and suppresses the maturation of specific tumor suppressor miRNAs under hypoxic conditions [69]. 

Ago2 is the catalytic protein part of the miRISC complex, serving as an additional regulator of mRNA stability [48]. Ago2 is regulated at the transcriptional and post-transcriptional levels by multiple mechanisms, including arginine methylation, Met1-linked linear ubiquitination, DDX21, and E-cadherin [70,71,72,73]. Nucleolin is a multifunctional protein found mainly in the nucleolus. It has roles in transcription, ribosome biogenesis, DNA replication, chromatin remodeling, apoptosis, and micropinocytosis [74]. Nucleolin works as a transcription factor and as a regulator through its interactions with other proteins. It promotes the maturation of specific miRNAs implicated in carcinogenesis cells [75].

## 6. Regulation of miRNA by Estrogens

Estrogens act on ERα and ERβ both on genomic and membrane-initiated, non-genomic levels. This review focuses on the genomic activity of ERs; the effects of estrogens on non-genomic levels have been reviewed sufficiently elsewhere [76,77]. ERs belong to the superfamily of nuclear receptors (NRs) [78]. Any NR has a generally outlined structural organization made up of six regions named A-F, each of which has a specific function that varies significantly amongst different receptors’ subtypes. They also exhibit a common structure, with four similar functional domains: the NH2-terminal regulatory domain (the A/B domain), which is quite variable compared to the highly conserved DNA binding domain (C domain), the hinge region (D domain), and the E domain, which contains a C terminal ligand-binding domain (LBD) [79,80]. 

E2 has a high affinity for binding to the LBD of ERs. Such binding forces ER to become more specific, as a transcription factor, to DNA regions that contain the following sequence: 5’-GGTCAnnnTGACC-3′, the so-called “estrogen-responsive element” (ERE) [81]. A group of factors called “pioneer factors” enhances the binding ERs to the ERE. These include FoxA1, PBX, TLE1, AP2g, and GATA3 [82,83,84]. They remodel compacted and condensed chromatin into open chromatin. The compaction of chromatin has been proven to stand as a barrier against transcription. Remodeling of the condensed chromatin increases the accessibility to the DNA and increases target gene transcription by allowing RNA polymerase II to act on DNA and form miRNAs [85].

## 7. miRNA and the Nervous System

The last step in the processing of miRNAs is the Dicer-mediated cleavage. This final step is considered the interface link between miRNA and its regulators (e.g., E2 and androgens) on one hand and neurons on the other. Any disruption in the dicer-mediated cleavage of pre-miRNAs will affect mature miRNA production, which could propagate a negative effect on both cortical neurogenesis and the embryonic development of the nervous system [86]. Several studies concluded that the disruption of mature miRNAs would probably affect the function of the nervous system by causing a reduction in neural progenitor cells’ proliferation, a delay in the cell cycle, a disturbance in neural migration, an induction of apoptosis by activation of caspase 3, the stimulation of astrocyte differentiation, and the inhibition of neuronal differentiation [86,87,88,89]. 

Four highly conserved and distinguishable processes constitute the general term “neurogenesis” or “neural maturation”. The first is axonal and neurite outgrowth, followed by dendritic growth and synaptogenesis, neuronal maturation, and finally, cell apoptosis. Each process is regulated by a specific miRNA or a subset of miRNAs [90].

### 7.1. miR-9 Activity and Regulation by Estrogens (Table 1)

The activity of E2 and its action on miRNA are mainly mediated by the two ER subtypes (ERα, ERβ), both of which are expressed in the ventral hippocampus [91,92]. E2 participates in miRNA processing through ERα’s interaction with the Dorsha complex. Many studies discussed the regulation of different miRNAs by steroid hormones. They showed that miR-9 expression could be regulated by E2. The availability of binding sites for ER within miR-9 promoters increases the possibility that E2 directly impacts miR-9 transcription [93].

In a study performed to assess the correlation between miR-9 and breast cancer progression, the authors observed high variability in miR-9 expression in human samples; this variability was strongly related to the expression of ERs, where ERα binds to Drosha during the maturation of miRNAs [94]. The ingenuity pathway analysis (IPA) further delineated a regulatory loop of miR-9-5p expression influenced by ERs [95,96,97]. 

The Nuclear Factor kappa-light-chain-enhancer of activated B-cells activates miR-9. Then, a negative feedback loop occurs where miR-9-5p directly targets the mRNA of ERα [93]. Another study assessed E2 regulation for miRNAs, especially in the brain. E2 played a critical role in expressing mature functional miRNA in the brain, which was age- and brain region-specific. The regulation of miR-9 expression by E2 exhibited similar age changes; the three-month-old rats showed that E2 reduces miR-9 expression levels in the dorsal hippocampus, with miR-9-3p expression increased in the same region. However, in rats aged 18 months or more, E2 continued reducing miR-9 expression, while the controlling effect of E2 on miR-9-3p could not be detected anymore [98].

Sirtuin 1 (SIRT1) is a protein deacetylase that has a role in aging, and is a target gene of E2 [99,100]. It partakes in many vital functions of the brain, such as energy balance, memory processing, and neuroprotection [101,102]. miR-9 expression is inversely correlated with that of SIRT1, in which the 3′ UTR region complements with the miR-9 sequence [103].

**Table 1:** Molecules interacting with miR-9 and estrogen.

Molecule	Interaction with miR-9	Reference
NF-Kβ	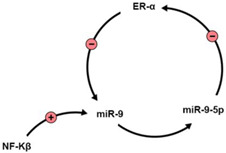	[93]
NEFH	miR-9 binds to the 3′UTR region of NEFH. miR-9-5p is significantly downregulated in ALS.	[104]
Serine Palmitoyltransferase [SPT]	SPT regulates Aβ in Alzheimer’s disease, and is correlated with miR-9 serum and cortical levels.	[105]
SIRT1	Negative correlation with miR-9 levels. SIRT1 gene is a target of E2.	[99,100,101,102,103]
E2	- Impacts expression via ERs on promotor regions or miR-9 and miR-9-5p. - Decreases miR-9 and increases miR-9-3p expression in dorsal hippocampus.	[95,96,97,98]
REST/CoREST	They regulate, and are regulated by miR-9. In HD, mutant huntingtin fails to regulate REST/CoREST, disrupting miR-9 activity.	[97,106,107]

### 7.2. miR-29 Family and Neurodegenerative Disorders (Table 2)

Principally, miRNAs have significant roles in gene expression regulation in different brain diseases and were suggested as therapeutic approaches in a wide range of human diseases because of their ability to deliver factors that would enhance repair mechanisms [108,109,110].

The miR-29 family is a group of miRNAs that is highly involved in developing mature and fully functional neurons. The miR-29 family consists of four transcripts; miR-29a, miR-29b-1, miR-29b-2 and miR-29c. Human miR-29a and miR-29b-1 are transcribed from chromosome 7, while miR-29b-2 and miR-29c are transcribed from chromosome 1. Both miR-29b-1 and 2 have the same mature sequence [111]. 

The first of the three sequences in this group is miR-29a. It has been suggested that miR-29a can be of great importance in the process of neural maturation, based on the observed upregulation of miR-29a gene expression during the differentiation of neural stem cells (NSCs). Infecting the NSCs with lentiviral-mediated miR-29a was associated with a significant increase in microtubule-associated protein 2 positive neurons, as well as a significant reduction in astrocytes. This is mediated through targeting the phosphatase and tensin homolog, commonly known as PTEN, which plays a vital role in the differentiation and growth of NSCs, making its deletion an essential step in optimizing neural maturation [112,113,114,115]. 

Other direct targets for miR-29a have been proposed, such as the expression of the protein Doublecortin, which restricts axonal branching [116]. By inhibiting this protein when it is overly expressed in mice’s cortical neurons, miR-29a increased the rates of axon branching in these cells [117]. Moreover, the widespread death of neurons was observed in mice following the knockdown of miR-29a using a chemically engineered oligonucleotide, i.e., locked nucleic acid antagomir (blockmir) called LNA29a/c. It is believed that the effect of miR-29a knockdown might be propagated through multiple mediators, like the voltage-dependent anion channel 1, a pro-apoptotic mediator that is inhibited by miR-29a [118,119]. miR-29b has been suggested as a rescue factor for neuronal cells, as it silences the pro-apoptotic BH3-only gene family [120]. In principle, miRNAs have significant roles in gene expression regulation in different brain diseases and were suggested as therapeutic approaches in a wide range of human diseases because of their ability to deliver factors that would enhance repair mechanisms.

The expression of miRNAs in embryonic and adult brains is controversial. It has been suggested that the miR-29 family is not expressed in the embryonic brain. However, further investigation showed that miR-29b is over-expressed in NSCs, and regulates embryonic proliferation and neurogenesis by targeting T-cell factor-mediated inhibitors, and the Wnt/β-catenin signaling pathway [121,122]. It was suggested that sex chromosomes might control miRNAs in neurodegenerative disorders [12,123]. In the brains of patients with neurological diseases such as Alzheimer’s disease (AD) or schizophrenia, only a few human studies have assessed sex differences in miRNA expression [124,125].

**Table 2:** Molecules interacting with miR-29.

Molecule	Interaction with miR-29	Reference
Doublecortin	miR-29a targets doublecortin expression, reducing axonal branching	[116]
Voltage-dependent anion channel 1	miR-29a regulates this molecule, reducing apoptosis	[118,119]
BH3-only family	miR-29b silences this proapoptotic gene family	[120]
Wnt/βcatenin signaling	miR-29b regulates this pathway, hereby affecting embryonic proliferation and neurogenesis	[121,122]
BACE	Negative correlation with miR-29a, miR-29b and miR-29c-3p expression in AD	[126]
DNA methyltransferase III beta (DNMT3B)	miR-29c acts on DNMT3B to reduce BDNF levels in AD invitro models.	[127]
Parkinsonism-associated Deglycase (PARK7)	miR-29 regulates this molecule. It is also regulated by estrogens and is implicated in PD pathology.	[128,129]
BcI2L2	BcI2L2 gene, which is antiapoptotic, is regulated by miR-29b.	[111,130,131,132]

### 7.3. Alzheimer’s Disease

The progression of AD is characterized by the accumulation of plaques made of short β-amyloid peptides. These peptides emerge from proteolytic cleavage of the β-amyloid precursor protein (APP) by a β-secretase known as the β-site APP-cleaving enzyme (BACE) [133,134]. It has been observed that miR-29a, miR-29b and miR-29c-3p have low expression levels in AD with abnormally high levels of BACE [126]. Despite the low expression levels, the miR-29 clad were found to be abnormally high in the CSF of two cohorts of AD patients [135,136], achieving 89% sensitivity and 70% specificity for the miR-29a [135], making it a potential candidate for future AD biomarker research [137]. However, some potentially contradicting evidence found low CSF miR-29c levels in AD patients. These were found to be linked to the decreased levels of BDNF expression, an effect that was posited to be mediated by DNA methyltransferase 3 through some in vitro experiments [127]. Besides, estrogens are essential in AD pathogenesis since they might decrease β-amyloid protein levels as a neuroprotective mechanism against the disease [125,138]. Pan and colleagues showed that estradiol’s neuroprotective effect against amyloid pathology in AD is potentially mediated by the miR-106b-5p/TXNIP axis in a neuroblastoma cell line [139], while another study showed that estradiol treatment on ovarectomized AD model mice slowed the pathological conformational changes of tau. This change was potentiated by the decreased expression of miR-218 [140]. Sedghi et al. showed that when levels of miR-29a are raised it produces a neuroprotective effect in the peripheral blood mononuclear cells of AD patients [141]. While this effect was realized using klotho and linagliptin treatment on isolated cells, it paves the road for testing other interventions with similar effects on the implicated pathways. 

### 7.4. Parkinson Disease

MiR-29a, miR-29b-1, and miR-29b-2 are over-expressed in the brains of Parkinson’s disease (PD) patients [142], while miR-29b-2-5p was under-expressed in another study [143]. miR-29 has significant roles in PD pathology, such as apoptosis and neuronal survival, motor function tuning, the immune response (by regulating T1 helper cells), and genetic modulation [144,145]. Inhibiting miR-29 expression in the mouse brain caused massive rates of neuronal death, especially in the hippocampus and cerebellum [119]. miR-29 also targets Parkinsonism Associated Deglycase, which is thought to be regulated by androgens and estrogens [128,129]. We believe that the role of miR-29 in the pathophysiology of PD needs further investigation in the clinical setting [144,146]. As for other miRNAs, Liu and colleagues used computational methods to study the differentially-expressed genes in PD patients. By constructing a miRNA-mRNA regulatory network, they found that has-miR-142 was the most vital miRNA in the network, carrying out its effects on GNAQ, TMTC2, KYNU, and BEND2 [147].

### 7.5. Huntington’s Disease

Pre-clinical studies done on HD showed a consistent downregulation of miR-9 and miR-29; this effect was constant across different animal and animal cell models [148]. One of miR-9/9*’s functions is regulating the function of REST/CoREST in the cell. Because REST/CoREST targets miR-9/9* as well, and because mutant huntingtin in HD patients fails to regulate the levels of REST in the cell, levels of REST become unusually high in the neurons of HD patients. Ultimately, this leads to the misregulation of gene expression in those neurons [106,107]. Another study found that miR-9*, but not miR-9 or miR-29b, were significantly downregulated in the peripheral leukocytes of HD patients compared to controls [97]. However, the level of miR-9* was not correlated to the UHDRS score in HD patients.

### 7.6. Other Brain Diseases

Owing to their potent antifibrotic effect, miR-29s have been strongly linked to the pathophysiology and management of stroke. At the infarction region, miR-29b levels were notably lower than in other healthy brain areas. Studies showed significant improvement in patients’ outcomes when utilizing a special approach to deliver a miR-29b mimic to combat the stroke-induced loss of miR-29b. It was also postulated that miR-29b levels were significantly increased during ischemic brain injuries. A recent study found that the overexpression of miR-29b reduced the neuronal cell death observed during brain ischemia. Conversely, the downregulation of miR-29b was accompanied by increased rates of neuronal cell death. It was proposed that miR-29b carries its action through inhibiting the expression of the antiapoptotic gene BcI2L2. Therefore, when BcI2L2 is overexpressed, it leads to increased neuronal cell survival [111,130,131,132]. 

The modulation of post-stroke-induced neurogenesis by miR-9, and Histone Deacetylase 4 was documented [149]. In brain neurogenic areas, miR-9 is widely expressed, which has ramifications for the differentiation of embryonic, and adult progenitor cells [150]. The effect of miR-9 in reducing neuronal apoptosis after ischemic stroke was investigated in a few studies [151,152]. Wei et al. found that miR-9 directly targets Bcl2l11, and that altering miR-9 causes changes to Bcl2l11 protein levels [151]. The authors concluded that miR-9 targets Bcl2l11 to facilitate cell apoptosis. Another study showed that miR-9 upregulation promoted neuronal survival, and regeneration following ischemic stroke [153]. The authors put forth a key mechanism by which HDAC4 inactivation positively regulated the expression of miR-9 and ameliorated ischemic insult in vitro. This may contribute to overcoming the hurdles hampering the adoption of miRNA-based therapeutics for ischemic stroke. A recent study by Wang et al. showed that, compared to healthy people, early-stage acute ischemic stroke patients had higher blood levels of miR-9-5p. Serum levels of miR-9-5p were significantly correlated with patient prognosis, with high concentrations being linked to unfavorable patient outcomes [154]. 

In gliomas, Wu et al. documented that elevated miR-9 expression might be involved in tumour progression, and proposed miR-9 as a valuable marker to predict the clinical prognosis of glioma patients, particularly those with advanced subtypes [155]. Another study by Tan et al. [156] showed that miR-9 is substantially expressed in glioma cells. miR-9 suppressed glioma cell growth, and increased migration by directly targeting cAMP-response element binding protein as well as neurofibromin 1. Further studies also documented that miR-9 plays an important role in glioma pathogenesis, and might be used as a prognostic marker, and possible therapeutic target for gliomas [157,158]. On the other hand, miR-9 overexpression significantly inhibited the growth of U87 glioma cells. The growth limitation was primarily attributable to the stimulation of apoptosis, which coincided with an increase in the Bax/Bcl-2 ratio [159]. miR-9 overexpression caused cell cycle arrest in U87 glioma cells at the G2/M checkpoint, and miR-9 inhibited the migration as well as invasion of U87 glioma cells [159]. In their study, Shi et al. investigated miR-29s as a tumor suppressor in gliomas using 187 human glioma specimens as well as 20 nontumoral brain specimens. The authors documented that when glioma grade and the Ki-67 index rose, the expression of miR-29a/b/c substantially decreased [160]. This highlights the importance of miR-29a/b/c and TRAF4 in predicting prognosis and their possible therapeutic role in malignant gliomas.

Research has gradually moved away from safeguarding neurons and toward investigating the combined effects of the neurovascular unit on traumatic brain injury (TBI) in recent years as a result of the ongoing investigation of the pathological process [161,162,163]. This shift highlights the necessity of angiogenesis for neurological functional recovery after TBI. By triggering the Hedgehog pathway, and enhancing the p-AKT expression, Wu et al. documented that miR-9-5p stimulates angiogenesis in the injured cerebral cortex. This highlights the possibility that miR-9-5p may be a useful therapeutic target for TBI. Mu et al. provided a new function for miR-29a in controlling NSC development and neurite outgrowth. They also provided a potential theoretical base for how NSC migration contributes to brain growth as well as damage repair [164]. By inhibiting NLRP3 expression and activation, miR-29a-5p mimics have been documented to protect against TBI-induced enhanced endothelial cell permeability, and BBB dysfunction [165].

## 8. Future Directions

Limited in silico research highlighted the possibility of interactions between the miR-29 family and estrogens in a neurodegenerative context [166,167]; such interactions have yet to be verified experimentally. Most of the studies reviewed here have been carried out in cell cultures, rats, or mice. There is a need to use other animal models, including non-human primates, to gain more reliable insights into the mechanisms highlighted in this review. Recent research showcased the prevalence of sex differences among neurodegeneration patients; such differences have been partly attributed to sex hormones, including estrogens [168,169,170], but have yet to be connected to miRNAs in a clinical setting. While the use of estrogen replacement therapy for treating Alzheimer’s [171], Parkinson’s [172,173,174,175], and Huntington’s [176] diseases was thoroughly tested in previous work, more precise therapies targeting miR-9/29 pathways need to be explored.

## 9. Conclusions

The miRNA–estrogen interaction has been studied thoroughly over the past few decades. The hypothesis that this relation is somehow related to developing some major neurological disorders constitutes a starting point for further clinical research within the same subject area. A proper understanding of the nature of this tri-axial system will contribute to the development of more advanced diagnostic techniques and more convenient treatment options in a variety of neurological disorders. We summarized the different regulation routes that estrogens impose on miRNAs, specifically miR-9 and miR-29. Furthermore, we overviewed the multiple effects for altering miRNAs’ transcription, maturation, and gene expression in different neural tissues. We then examined the possible benefits for this hormone-induced transcriptional modification in some neurological conditions using the currently available evidence.

## Data Availability

Not applicable.
